# Educational interventions in health services and oral health: systematic review

**DOI:** 10.11606/S1518-8787.2018052000109

**Published:** 2018-05-03

**Authors:** Aryane Marques Menegaz, Alexandre Emídio Ribeiro Silva, Andreia Morales Cascaes

**Affiliations:** I Universidade Federal de Pelotas. Faculdade de Odontologia. Programa de Pós-Graduação em Odontologia. Pelotas, RS, Brasil; II Universidade Federal de Pelotas. Faculdade de Odontologia. Departamento de Odontologia Social e Preventiva. Pelotas, RS, Brasil

**Keywords:** Health Education, Dental, Evaluation of the Efficacy-Effectiveness of Interventions, Dental Health Services, Review, Educação em Saúde Bucal, Avaliação de Eficácia-Efetividade de Intervenções, Serviços de Saúde Bucal, Revisão

## Abstract

**OBJECTIVE:**

To analyze the effectiveness of educational interventions performed in health services in the improvement of clinical behaviors and outcomes in oral health.

**METHODS:**

We have carried out a systematic review of the literature searching the PubMed, Lilacs, and SciELO databases. We have included studies that have investigated interventions performed by health professionals working in health services and who have used educational actions as main approach to improve behavioral and clinical outcomes in oral health.

**RESULTS:**

The search amounted to 832 articles and 14 of them met all the inclusion criteria. Five studies have only exclusively evaluated the effectiveness of interventions on caries reduction, three have exceptionally evaluated oral health behaviors, and the other articles have evaluated the effectiveness of interventions for both clinical outcomes (dental caries and periodontal conditions) and behaviors in oral health. Most of the studies (n = 9) were based on randomized controlled trials; the other ones have evaluated before and after the intervention. Five studies have reported a significant reduction of dental caries, and five of the six studies evaluating behavioral outcomes have found some positive change.

**CONCLUSIONS:**

Most studies evaluating behavioral and periodontal outcomes have shown significant improvements in favor of interventions. All studies evaluating caries have shown a reduction in new lesions or cases of the disease in the groups receiving the interventions, although only five of the eleven articles have found a statistically significant difference. Educational interventions carried out by health professionals in the context of their practice have the potential to promote oral health in the population.

## RESUMO

**OBJETIVO:**

Analisar a efetividade de intervenções educativas realizadas em serviços de saúde na melhoria de comportamentos e desfechos clínicos em saúde bucal.

**MÉTODOS:**

Foi realizada uma revisão sistemática da literatura com busca nas bases de dados PubMed, Lilacs e SciELO. Foram incluídos estudos que investigaram intervenções realizadas por profissionais de saúde atuantes em serviços de saúde e que utilizaram como principal abordagem ações de educação com a finalidade de melhorar desfechos comportamentais e clínicos em saúde bucal.

**RESULTADOS:**

A busca totalizou 832 artigos e 14 preencheram todos os critérios de inclusão. Cinco estudos verificaram exclusivamente a efetividade das intervenções na redução de cárie, três avaliaram excepcionalmente comportamentos em saúde bucal, os demais artigos julgaram a efetividade das intervenções tanto para desfechos clínicos (cárie dentária e condições periodontais) quanto para comportamentos em saúde bucal. A maioria dos estudos (n = 9) foi baseada em ensaios randomizados e controlados, os demais avaliaram o antes e depois da intervenção. Cinco estudos reportaram redução significativa de cárie dentária, e cinco dos seis estudos que avaliaram desfechos comportamentais encontraram alguma mudança positiva.

**CONCLUSÕES:**

A maioria dos estudos que avaliaram desfechos comportamentais e periodontais mostrou melhorias significativas a favor das intervenções. Todos os estudos que avaliaram cárie mostraram a diminuição de novas lesões ou casos da doença nos grupos que receberam as intervenções, embora apenas cinco dos 11 artigos tenham encontrado uma diferença estatisticamente significativa. As intervenções educativas realizadas por profissionais de saúde no contexto de sua prática apresentam potencial em promover a saúde bucal da população.

## INTRODUCTION

Oral diseases are considered an important public health problem not only because of their high prevalence^22^ ^–^ ^24^ but also because they negatively affect the quality of life of individuals and have high costs for their treatment^31^. Data from the global study “Global Burden of Disease Study 2010” revealed that approximately 3.9 billion persons are affected by oral diseases^34^. Untreated caries in permanent teeth was the health condition with the highest overall prevalence (35% for all ages combined)^34^.

The fact that oral diseases are largely preventable and yet present high prevalence is worrying. Healthy behaviors, such as daily tooth brushing, regular contact with fluoride sources^35^, and controlled consumption of sugar^49^ are the most effective ways to prevent major oral diseases, as well as reduce costs for health services and society^47^. The strong social and behavioral character of these diseases exposes the importance of the implementation of educational interventions for the appropriation of self-knowledge about the health-disease process, stimulating the autonomy and change in health behaviors leading to prevention^1^.

Systematic and narrative reviews of studies evaluating the effect of educational interventions on the improvement of oral health, published between 1982 and 2013, have been previously conducted. A narrative review has sought to identify evidence of strategies for the promotion of oral health in its evaluation, including three systematic reviews and two narratives on the effectiveness of educational interventions in improving oral health, based on studies published between 1982 and 1996^59^. The five reviews analyzed present similar results regarding the short-term positive effects in knowledge but which are limited in relation to behaviors and clinical conditions^59^. We highlight that the design, methodology, and evaluation of the studies are criticized as being of low quality^59^.

Twetman^55^ has systematically reviewed 22 studies on several types of intervention for caries prevention in children up to three years of age, published between 1998 and 2007. Educational interventions have been reported in five studies. The author has found that two low quality studies had zero effects, whereas three studies of average quality identified a lower incidence of caries in the group that received health education when compared to the group that did not receive it. The author states that the most effective studies were those conducted in the context of health services or that used motivational interviewing as an educational approach.

Lemkuhl et al.^30^ have carried out a narrative review of the literature that has analyzed the impact of 37 studies on educational interventions for the improvement of clinical diseases in oral health, published between 2003 and 2013. Inconsistent effects were found for plaque reduction, gingival bleeding, increased caries, and dental calculus. However, studies with more contact with the target public had the greatest magnitudes of positive effect in the investigated outcomes. The methodological quality of the recently published studies is still poor, according to the analysis of the authors. In addition, most of the educational approaches used are essentially based on an individual and traditional pedagogical model. These approaches are criticized for disregarding the social, interpersonal, and context relations of health services.

Studies that evaluate the effect of educational interventions integrated into health services and aimed at improving oral health have not been systematically reviewed. None of the reviews previously presented have classified interventions integrated into health services as an important category of analysis. Only Twetman^55^ has observed that studies of this nature present greater potential for the prevention of caries in children. In the perspective of integrated interventions in health services, different health professionals promote actions that increase the ability to obtain positive effects on the health of individuals and communities^62^. Health education is listed as one of the central elements in the success of this type of intervention in several health areas^14^. Regarding oral health, contact with individuals and communities should be viewed by any health professional as an opportunity for educational work, aiming to contribute to the improvement of the oral health conditions of the population, especially in places with restricted access to the dentist.

Knowledge of the effectiveness of educational interventions for oral health helps in the identification of the best strategies to be applied in the context of health practices. Given the – educational – nature of the intervention, the scientific validity of most of the clinical trials analyzed in previous reviews is questionable. Unlike classical clinical interventions, carried out under optimum conditions to produce the expected effect, success in educational interventions depends on a range of players involved in the implementation and evaluation. The desired changes in individuals must be followed up by changes in the professional and organizational practices of health services. Such services should be focused on an integrated perspective and offer continuous support to users, using health education as a tool to achieve this purpose. The understanding on how this type of intervention should work, in the real world, is crucial to achieve improvements in oral health. Given this context, this systematic review was carried out to answer the question: Are the educational interventions integrated into health services effective in modifying oral health behaviors or in preventing oral diseases?

## METHODS

The guidelines of the PRISMA protocol were followed for the reporting of this review^41^. The review is registered on the PROSPERO platform (CRD42016052112).

We have carried out a systematic review of the literature on the PubMed, Lilacs, and SciELO databases. There were no date or language limits. We checked the gray literature and the references of the selected articles to find additional studies that were not identified in the searches.

The term used in the search combined the following terms in the Medical Subject Headings (MeSH) and their corresponding words in the Health Sciences Descriptors (DeCS): (((“Health Promotion”[Mesh] OR “Health Education, Dental”[Mesh]) OR “Health Education”[Mesh] OR “Community Health Workers/education”[Mesh] OR “Health Personnel/education”[Mesh] OR “Education, Medical, Continuing”[Mesh] OR “Pediatrics/education”[Mesh] OR “Preventive Dentistry”[Mesh])) AND “Oral Health”[All Fields] AND (“Clinical Trial”[Publication Type] OR “Intervention Studies”[All Fields] OR “Evaluation Studies”[Publication Type] OR “Program Evaluation”[Mesh]). We generated a database with the research results using the EndNote X7 tool.

We included in this review: (i) intervention studies, with or without randomization, with or without a control group, covering a population of any age group; (ii) educational interventions, covering general or oral health, performed exclusively by health professionals, who performed their work in public or private health services of any context (e.g.: hospitals, primary health care centers, reference services in universities, medical and dental clinics); (iii) studies evaluating the outcome of improvements in oral health-related behaviors (the main outcomes were: daily brushing at least twice a day, use of baby bottle, consumption of sweets, use of dental services) or evaluating outcomes under clinical conditions of oral health (caries and periodontal conditions were the only ones found).

We excluded the studies: (i) in which preventive or curative treatment was the main intervention, as our focus was to evaluate the effect of educational interventions; (ii) whose objective was to evaluate knowledge acquisition and effects in the practice of students or professionals, after training to exercise health education actions and not their effect on the target population; (iii) in schools, with the exception of those explicitly implemented by health professionals; iv) with very small samples (less than or equal to 20 participants).

The selection of the articles began with the reading of the titles and abstracts carried out independently by two of the authors (AMM and AMC). The final decision was based on a third reviewer in cases of disagreement (AERS). After selection, the following data related to the characteristics of the studies were extracted by two of the authors (AMM and AMC): author, sample, place of the study (developed or developing countries), type of study (randomized and controlled trial or before and after), type of health service (primary care health center, hospitals, medical or dental clinic), target audience, type of educational resource or strategy used, professional who implemented the intervention (dentist, physician, nurse, community health agent, more than one type of professional), dosage of the intervention (1 to 3, 4 to 12 contacts with the target public), maximum evaluation time in months (2 to 6, 7 to 12, 13 to 24, 36 to 60), follow-up rate of more than 80% (no or yes), and type of outcome (clinical, behavioral or clinical, and behavioral), besides the results obtained in the studies.

We presented the results for the outcomes as such in the articles and, subsequently, we calculated an estimate of the relative impact of the interventions on the outcomes, according to an approach previously presented in the literature^30^. We performed the following calculations in each group: final result of the outcome evaluation (FR) subtracted from the initial result (IR), divided by the initial result and multiplied by one hundred: ((FR - IR) / IR) × 100. When the study had a control group, we compared the percentage values of the intervention group to the control group, using a calculation similar to the one mentioned above. In this way, we estimated the magnitude of the decreases or increases in the outcomes of the intervention group in relation to the control group in percentage terms.

Two of the authors evaluated the quality of the articles (AMM and AMC). The two authors initially discussed the quality evaluation criteria, and the evaluation was done together. The differences were debated and we took the final decision by consensus. We evaluated the quality of the studies with the instrument proposed by Downs and Black^15^, originally with 27 questions related to the quality of the information present in the article, external validity, internal validity (bias and confounding), and statistical power, which gives a score ranging from zero to 28. The issue about the attempted to blind the subjects to exposure was excluded, as it does not apply to the type of intervention performed. Therefore, the score of the articles could range from zero to 27. We classified each study according to the quality of the evidence as excellent (24 to 27), good (20 to 23), reasonable (15 to 19), or poor or limited (14 or less), according to criteria used in another review^7^. The quality of the evidence was not an exclusion factor, since we considered it important to evaluate all the available evidence about the subject and relate it to the results found.

All data were tabulated in worksheets of the Excel^®^ 2013 program. We calculated the absolute and relative frequencies and performed the estimates of the relative impact of interventions.

## RESULTS

The searches resulted in a total of 832 articles, which became 830 articles after removing the duplicates. In this first stage, we excluded 800 articles after reading the title and abstract, thus leaving us with 30 articles for the reading of the full text. Subsequently, we excluded one study because it presented a small sample (n = 20)^3^, nine studies were not implemented by health professionals working in the service^12^ ^,^ ^13^ ^,^ ^17^ ^,^ ^21^ ^,^ ^27^ ^,^ ^28^ ^,^ ^33^ ^,^ ^45^ ^,^ ^51^, four studies had the preventive and curative treatment as the main intervention^16^ ^,^ ^24^ ^,^ ^38^ ^,^ ^44^, one study described the protocol and did not evaluate effects^53^, and one study was a duplicate^11^. At the end, fourteen studies considered all the criteria (Figure 1) and were included in the qualitative synthesis^2^ ^,^ ^8^ ^–^ ^10^ ^,^ ^19^ ^,^ ^22^ ^,^ ^29^ ^,^ ^39^ ^,^ ^42^ ^,^ ^46^ ^,^ ^54^ ^,^ ^57^ ^,^ ^58^ ^,^ ^61^.

**Figure:**
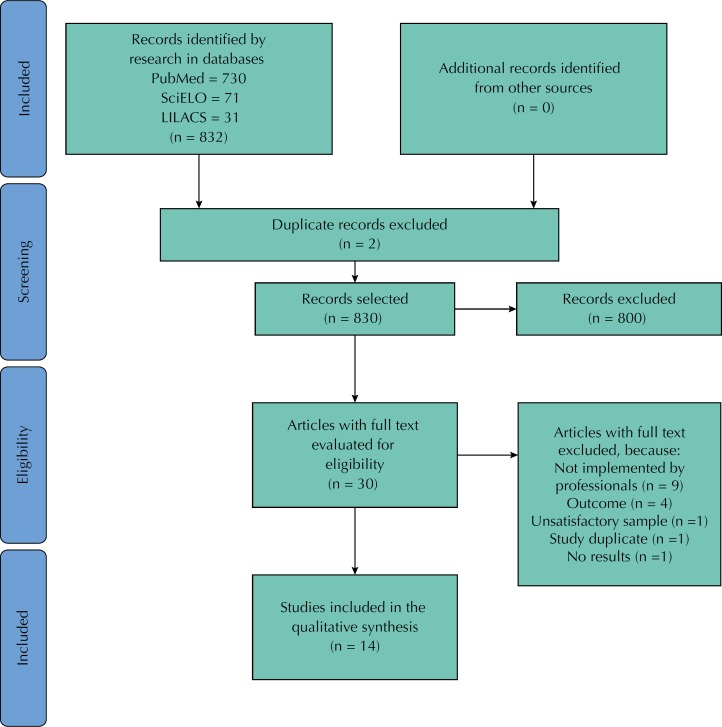
Flowchart for the selection of studies Adapted from the PRISMA statement^41^.

Table 1 summarizes the main characteristics of the studies included in the review. Half of the studies were carried out in developing countries^8^ ^,^ ^19^ ^,^ ^22^ ^,^ ^39^ ^,^ ^42^ ^,^ ^46^ ^,^ ^57^ and most (n = 9) included parents and their children as target population^2^ ^,^ ^10^ ^,^ ^22^ ^,^ ^39^ ^,^ ^46^ ^,^ ^54^ ^,^ ^57^ ^,^ ^58^ ^,^ ^61^, with age varying from zero to eight years. Most studies (n = 8) were based on randomized controlled trials^2^ ^,^ ^8^ ^–^ ^10^ ^,^ ^39^ ^,^ ^46^ ^,^ ^57^ ^,^ ^61^ and seven were carried out in primary health care centers^8^ ^,^ ^10^ ^,^ ^19^ ^,^ ^39^ ^,^ ^46^ ^,^ ^57^ ^,^ ^61^. Only one study did not offer verbal advice/guidance as an educational strategy^9^. One study offered only healthy eating guidelines^8^; the others offered, mainly, oral hygiene guidelines. The dosages of the intervention (i.e., the number of times the intervention was performed in a given population in the proposed time period) varied from one to twelve contacts with the target audience, and half of the studies had up to three contacts^9^ ^,^ ^19^ ^,^ ^29^ ^,^ ^39^ ^,^ ^57^ ^,^ ^58^ ^,^ ^61^. Only five studies had a follow-up rate above 80%^2^ ^,^ ^9^ ^,^ ^22^ ^,^ ^46^ ^,^ ^54^.

**Table 1:** Description of studies included in the review. (n = 14)

Characteristics of the studies		n	%
Place of the study			
	Developed countries	7	50.0
	Developing countries	7	50.0
Type of study			
	Randomized and controlled test	9	64.2
	Before and after	5	35.8
Type of health service			
	Primary health care center	7	50.0
	Ontological clinic	3	21.4
	Hospital	3	21.4
	Medical clinic	1	7.2
Target audience			
	Parents and their children	9	64.2
	Pregnant women	3	21.4
	Children	1	7.2
	Women who use public services	1	7.2
Type of educational resource or strategy used*			
	Oral advice/Guidance	13	92.9
	Giving out handouts/Pamphlets	7	50.0
	Giving out toothbrushes and toothpastes	6	42.8
	Posters in health services	2	14.3
	Demonstration of brushing in macromodels	2	14.3
	Sending of postcards by mail	1	7.2
	Video demonstration	1	7.2
	Photo album demonstration	1	7.2
	Giving out sippy cups	1	7.2
	Phone calls	1	7.2
Professional who implemented the intervention			
	More than on professional	6	42.8
	Dentist	3	21.4
	Community health agent	3	21.4
	Physician	1	7.2
	Nurse	1	7.2
Dosage of the intervention (number of contacts)			
	1 to 3	7	50.0
	4 to 12	5	35.8
	Not informed	2	14.2
Maximum time for evaluation (months)			
	2 to 6	3	21.4
	7 to 12	5	35.8
	13 to 24	1	7.2
	36 to 60	3	21.4
	Not informed	2	14.2
Follow-up rate above 80%			
	No	7	50.0
	Yes	5	35.8
	Not informed	2	14.2
Outcomes			
	Clinical	6	42.8
	Behavioral	3	21.4
	Clinical and behavioral	5	35.8

* Some studies have used more than one type of resource; the percentage values shown are individual for each type of resource.

The time of follow-up of the interventions varied greatly, and the most frequent was between seven and 12 months^9^ ^,^ ^19^ ^,^ ^22^ ^,^ ^46^ ^,^ ^57^. Five studies have only exclusively evaluated the effectiveness of the intervention on caries reduction ^8^ ^,^ ^29^ ^,^ ^39^ ^,^ ^42^ ^,^ ^61^, three have evaluated only oral health behaviors^9^ ^,^ ^19^ ^,^ ^54^, and the other ones have judged the effectiveness of the interventions for both clinical outcomes (dental caries, dental plaque, periodontal diseases) and behavior^2^ ^,^ ^10^ ^,^ ^22^ ^,^ ^46^ ^,^ ^57^ ^,^ ^58^.

The quality evaluation of the studies is expressed in Table 2. The average total score was 17.6 points (SD = 4.2) according to the instrument of Downs and Black^15^. The minimum score was 11 points^42^, and only two studies scored 25 points^8^ ^,^ ^39^, which were considered excellent. Two interventions were considered good^10^ ^,^ ^57^ and seven were considered of reasonable quality^2^ ^,^ ^9^ ^,^ ^19^ ^,^ ^29^ ^,^ ^46^ ^,^ ^54^ ^,^ ^58^. The evaluation according to the items of the instrument identified greater methodological problems related to confounding, external validity, and reporting.

**Table 2:** Evaluation of the quality of the interventions, according to the criteria of Downs and Black^15^.

Authors, year	Report	External validity	Bias*	Confounding	Power	Sum
	(0 to 10)	(0 to 3)	(0 to 6)	(0 to 6)	(0 to 1)	(0 to 26)
Chaffee et al.^8^ (2013)	10	3	5	6	1	25
Mohebbi et al.^39^ (2007)	9	3	6	6	1	25
Vachirarojpsian et al.^57^ (2005)	8	3	6	5	0	22
Davies et al.^10^ (2005)	8	3	5	3	1	20
Raj et al.^46^ (2003)	8	3	5	2	1	19
Blinkhorn et al.^2^ (2003)	6	1	6	3	1	17
Cibuka et al.^9^ (2011)	9	0	5	2	1	17
Frazão et al.^19^ (2009)	7	3	4	2	1	17
Wagner et al.^58^ (2013)	7	3	3	3	1	17
Strippel et al.^54^ (2010)	8	0	4	3	1	16
Larsen et al.^29^ (2016)	8	1	4	1	1	15
Gauba et al.^22^ (2016)	8	0	4	1	1	13
Whittle et al.^61^ (2008)	5	0	5	2	1	13
Moskovitz et al.^42^ (2009)	7	0	2	2	0	11
Average (SD)	7.7 (1.3)	1.6 (1.4)	4.6 (1.2)	2.9 (1.6)	0.9 (0.4)	17.6 (4.2)

* Question 14 of the Downs and Black instrument was excluded.

Table 3 includes the results of the interventions on the oral health behaviors analyzed. Six studies evaluated daily brushing at least twice a day and most (n = 5) presented positive results in favor of the interventions^9^ ^,^ ^10^ ^,^ ^46^ ^,^ ^57^ ^,^ ^58^, with magnitudes of effect ranging from 11.0%^9^ to 141.5%^46^. We also found significant differences regarding the use of baby bottle and consumption of sweets for most studies; five of the six studies have demonstrated improvement in these outcomes in the groups receiving the interventions^9^ ^,^ ^10^ ^,^ ^22^ ^,^ ^54^ ^,^ ^57^, whose magnitudes of effect ranged from 7.6%^54^ to 83.3%^10^. Finally, the three studies that proposed the increase in the use of dental services found positive effects for the intervention^9^ ^,^ ^19^ ^,^ ^58^. In two of the them^9^ ^,^ ^19^, we observed the highest magnitudes of effect in relation to all the behaviors evaluated.

**Table 3:** Results of the interventions on the main behaviors in oral health.

Authors, year	Control group	Intervention group	p	Relative impact of intervention (%)
Daily brushing at least twice a day				
Cibulka et al.^9^ (2011)	I: NI F:NI	I: average score 1.8 (SD = 0.62) F: average score 2.0 (SD = 0.69)	0.013	11.0
Davies et al.^10^ (2005)	I: NI F: 34%	I: NI F: 52%	< 0.001	52.9
Frazão et al.^19^ (2009)	-	I: 73% F: 88%	> 0.05	20.6
Raj et al.^46^ (2013)	-	I: 4.1% F: 9.9%	< 0.001	141.5
Vachirarojpisan et al.^57^ (2005)	I: NI F: 26.7%	I: NI F: 41.8%	< 0.001	56.6
Wagner et al.^58^ (2013)	They conclude that mothers who participated in the intervention started brushing the teeth of their children earlier and more frequently using fluoride toothpaste. Results in numbers are not shown.			
Use of baby bottle				
Davies et al.^10^ (2005)	Stopped using baby bottle			
	I: NI F: 18%	I: NI F: 33%	0.04	83.3
Strippel et al.^54^ (2010)	Frequency of bottle feeding with cariogenic content during the day			
	I: NI F:41%	I: NI F: 32%	< 0.001	-21.9
Vachirarojpisan et al.^57^ (2005)	Sleep using a bottle			
	I: 44% F: 35.1%	I: 43.7% F: 40.4%	> 0.05	*
Consumption of sweets				
Cibulka et al.^9^ (2011)	Intake of soft drinks more than twice a day			
	I: 21% F: 23%	I: 18% F: 11%	< 0.05	-48.7
Gauba et al.^22^ (2016)	Consumption of a cariogenic diet			
	-	I: Average 8.47 (SD = 5.18) F: Average 2.37 (SD = 2.23)	< 0.001	-72.1
Strippel et al.^54^ (2010)	Introduction of sugars in food at 7 months			
	I: NI 32%	I: NI F: 24%	< 0.001	-25.0
Vachirarojpisan et al.^57^ (2005)	Frequent consumption of sugary drinks during the day at 24 months of age			
	I: NI F: 66%	I: NI F: 61%	0.013	-7.6
	Consumption of sweets between meals			
	I: 92.1% F: 90.6%	I: 88.3% F: 91.5%	> 0.05	*
Use of dental services				
Cibulka et al.^9^ (2011)	Went to the dentist last year			
	I: 30.1% F: 32.9%	I: 27.4% F: 56.9%	0.006	1.061.0
Frazão et al.^19^ (2009)	Frequent or very frequent use of dental services			
	-	I: 25.3% F: 57.1%	< 0.001	125.7
Wagner et al.^58^ (2013)	They conclude that mothers who participated in the intervention reported more use of dental services in those who received the intervention. Results in numbers are not shown.			

* The change was greater in the control group than in the intervention group.

The results regarding a decrease in oral diseases and disorders are shown in Table 4. Of the 11 studies evaluating the prevention of new lesions/cases of caries, five presented significant differences at the end of their interventions^10^ ^,^ ^22^ ^,^ ^29^ ^,^ ^42^ ^,^ ^58^, with magnitudes of effect varying from 31.6%^10^ to 481.6%^22^. Although most interventions (n = 6) have not presented significant differences for caries disease, all studies have shown a decrease in dental caries in the groups that received the interventions. Furthermore, two other studies^2^ ^,^ ^46^ addressed the clinical outcome of dental plaque and one the outcome of dental calculus^46^, with positive results.

**Table 4:** Results of the interventions for clinical outcomes

Authors, year	Index	Control group	Intervention group	p	Relative impact of intervention (%)
Dental caries					
Blinkhorn et al.^2^ (2003)	dmft	I: 2.2 (SD 2.3) F: 3.2 (SD 2.8)	I: 2.0 (SD 2.2) F: 2.6 (SD 2.6)	0.21	-33.3
Chaffee et al.^8^ (2013)	Average dmfs (with white spots)	I: zero F: 3.6 (SD 6.9)	I: zero F: 2.8 (SD 5.4)	0.25	-22.2
	Average of the decayed component of the dmfs	I: zero F: 3.0 (SD 6.8)	I: zero F: 2.1 (SD 5.0)	0.18	-30.0
Davies et al.^10^ (2005)	Average dmft	I: NI F: 1.7	I: NI F: 1.1	< 0.001	-35.3
	Average dmfs	I: NI F: 3.8	I: NI F: 2.6	0.008	-31.6
Gauba et al.^22^ (2016)	They have not used the index. They were based on their own criteria and characterized it as “chance to avoid new cavities”	-	I: Average 12.5 (SD 13.5) F: Average 72.7 (SD 14.4)	< 0.001	481.6
Larsen et al.^29^ (2016)	They have not used the index. Presence or absence of dental caries	I: zero F: 29 children without dental caries (69%)	I: zero F: 19 children without dental caries (39%)	0.015	-43.5
Mohebbi et al.^39^ (2007)	Average of enamel caries WHO criteria (1997)	I: 0.08 (SD 0.4) F: 0.48 (SD 1.0)	Group A I: 0.25 (SD 0.7) F: 0.15 (SD 0.5)	0.283	Group A -108.0
			Group B I: 0.25 (SD 0.7) F: 0.35 (SD 1.0)		Group B -92.0
	Average of dentin caries WHO criteria (1997)	I: 0.03 (SD 0.25) F: 0.23 (SD 0.95)	Group A I: 0.04 (SD 0.2) F: 0.02 (SD 0.2)	0.719	Group A -107.6
			Group B I: 0.14 (SD 0.8) F: 0.12 (SD 0.3)		Group B -102.1
Moskovitz, et al.^42^ (2009)	Average of the decayed component of the DMFT	I: NI F: 5.9	I: NI F: 3.2	< 0.001	-45.8
	Average DMFT	I: NI F: 7.5	I: NI F: 6.8	> 0.05	-9.3
Raj et al.^46^ (2013)	Average dmft	-	I: 2.1 (SD 3.2) F: 1.9 (SD 1.4)	0.060	-9.5
Vachirarojpisan et al.^57^ (2005)	Cavitated and non-cavitated caries lesions*	I: 1.7 (SD 2.6) F: 7.7 (SD 5.2)	I: 1.9 (SD 2.7) F: 7.8 (SD 5.0)	> 0.05	-11.4
Wagner et al.^58^ (2013)	Average dmfs	I: zero F: 5.2 (SD 6.4)	I: zero F: 3.2 (SD 7.4)	< 0.05	-38.5
	Average dmft	I: zero F: 2.4 (SD 4.1)	I: zero F: 1.5 (SD 2.5)	< 0.05	-37.5
Whittle et al.^61^ (2008)	Average dmfs	I: zero F: 2.2 (36 months)	I: zero F: 2.0 (36 months)	> 0.05	-15.2
		I: zero F: 4.8 (60 months)	I: zero F: 4.0 (60 months)		
Periodontal conditions					
Blinkhorn et al.^2^ (2003)	Dental plaque - NI Index	I: NI F: 61%	I: NI F: 53%	0.16	-13.1
Raj et al.^46^ (2013)	Dental plaque - NI Index	-	I: 75.5% F: 66.5%	< 0.001	-11.9
	Gingival bleeding	-	I: 1.7% F: 2.2%	0.671	29.4
	Calculus	-	I: 78.3% F: 54.1%	< 0.001	-30.9

I: initial exam; F: final exam; NI: not informed; dmfs: surface index of decayed deciduous teeth extracted, and restored due to caries; dmft: index of decayed deciduous teeth extracted, and restored due to caries; DMFT: index of permanent teeth decayed, removed, and restored due to caries

* Criteria of Drury et al. (1999).

## DISCUSSION

The results of this review show that educational interventions promote changes in behaviors related to oral health. The magnitudes of effect of the interventions for these outcomes were decreasing in the following order: frequent use of dental services, adequate oral hygiene, and reduction in the consumption of sweets. Evidence of effectiveness for some periodontal outcomes, such as dental plaque and calculus, is limited to only two studies^2^ ^,^ ^46^, of reasonable quality, which have identified significant improvements. Although all educational interventions have observed a reduction in new lesions/cases of caries, only five studies, out of a total of 11, have reported these differences as significant. Among these five studies, only one was classified as with good methodological quality^10^.

The estimation of the magnitude of effects varied greatly between outcomes of the same category. Unlike other types of intervention, in which the outcome is estimated in terms of mortality rate or well-defined morbidity cut-off points, educational interventions have no ideal target parameter of change or magnitude of desired and relevant effect for public health. Studies could incorporate combined analyses about the benefits and associated costs or define the outcomes that represent the number of individuals to be treated for a favorable outcome. Approaches such as this contribute to reduce the existing limitation in the interpretation of the results of this type of intervention, as well as narrow the gaps between subjectivity and science.

The quality of the evidence of most studies was classified as poor/limited or reasonable. The main problems that led to this classification are related to: the type of design (before and after); insufficient description of the definition and validity of outcomes, especially outcomes of behavioral nature; lack of detail in the description of the interventions; lack of follow-up and fidelity in the implementation of protocols; high loss of follow-up and lack of reporting of the characteristics of the lost participants; lack of description of the population that originated the sample, as well as the criteria used in the selection; and lack of sample calculations and problems in the analysis and presentation of the results.

The positive effect on oral health behaviors may be an indication of the success of these interventions for disease prevention, since the first step in maintaining oral health is to build healthy behaviors. The great effect observed in the use of dental services reflects the result of interventions that linked strategies to facilitate access to the dentist, indicating the importance of interventions integrated to the health services for individuals. In this sense, the use of health services is not only an individual behavior, but a reflection of the context of professional practices and organization of services. Services that have a good organization of access and that work from an integrated health perspective increase the preventive use of the population. Individuals who use health services regularly have a greater opportunity to receive early diagnosis, acquire knowledge, and modify behaviors for their health, reducing the probability of developing health problems^4^ ^,^ ^56^.

The measurement of hygiene behaviors and the consumption of sweets are carried out by self-reported questions, which are liable to errors of information. In addition, such behaviors are usually evaluated with questions that have limited validity^6^. These self-reported questions and the nature of the educational intervention may reflect the knowledge acquired rather than a real change in behavior. The use of measures or instruments valid to measure behaviors, as well as their combination with the evaluation of clinical outcomes, contribute to the understanding of the effects of these types of interventions.

In order to have positive effects on clinical outcomes for oral health, such as caries, some methodological aspects of the studies should be taken into account. The use of insufficient samples, the small number of contacts with the target population, and the follow-up time of less than 12 months may have led to the lack of effect in many of the studies. Evidence indicates that the time for caries progression varies according to age and it can be approximately 6 to 12 months in young children and 24 to 36 months in adults^18^ ^,^ ^50^. However, in order to identify significant differences between groups, an appropriate sample calculation needs to be carried out, considering the number and size of clusters^25^ in the case of community interventions. The research studies tended to present sample calculations without considering the level of clusters of the participants, limiting the internal validity of the findings^40^ ^,^ ^48^.

The simple comparison of the groups by proportion and average tests does not ensure the significance of the results reported in the studies. With exception^8^, the studies did not present any evaluation of the rates of change and the relative effects of the interventions on the outcomes. The relative impacts of the interventions, calculated by us, are only estimates, in percentage terms, of the magnitude of the effects. The appropriate calculation of the measures of relative risk considers the rates of change in the groups, which depend on the time of follow-up and the number of individuals who started and finished the study. We suggest that future studies should consider these calculations in the evaluation of their results, presenting more real estimates of the effects of the interventions.

The dosage of the intervention, that is, the number of contacts with the target population, is another important factor for success in changing behaviors and consequent reduction of diseases. As discussed previously in the literature, educational interventions with at least four contacts with the target audience have a greater chance of success^7^ ^,^ ^30^. The evidence found in this review does not make clear what would be the minimum number of contacts needed to observe the desired effects. The relation between dosage and effects varied widely and we could not detect these differences among the interventions.

The latest evidence available in the literature on the effectiveness of educational interventions^23^ ^,^ ^26^, as well as this review, suggest the need for stricter scientific standards in monitoring interventions. In the studies evaluated in this review, we observed that the lack of evaluation of the implementation of interventions and the lack of clear theoretical models hindered the understanding of how interventions work or should work. Special attention should be given to the fidelity of the interventions, that is, to the extent to which an intervention adheres to the original model and its evaluation^52^. The greater the fidelity to the protocol of the intervention, the more positive is the result in the outcomes^5^ ^,^ ^36^. The fidelity of an intervention can be measured by different methods: quantitative and qualitative strategies, specific model scales, checklists with the key elements of the intervention, interviews with professionals or patients, focus groups, patient records, video analysis of the intervention, and phone interviews^5^.

In summary, the results included in this systematic review are difficult to compare, i.e., the type of educational intervention that is most effective remains unclear, since protocols differ greatly among the studies. The types of educational approach presented great variability; however, we detected that interventions with more than one associated method (video session, printed leaflet with guidelines, verbal guidance, and brushing practices) showed significant improvements in the reduction of dental caries^10^ ^,^ ^42^ ^,^ ^58^. However, more important than the pedagogical resource is the quality of the educational approaches, which should be customized according to the sociocultural context and preferably based on behavioral theories^60^.

Educational interventions integrated to health services become relevant to strengthen health systems, as they increase the chance of sustainability and continued support, resulting in beneficial long-term effects^62^. However, to reach these results, educational approaches need to be qualified, as those used by most health professionals have been portrayed as being traditional in many of the studies analyzed. Approaches based only on the transmission of knowledge have been severely criticized, since they transfer to individuals the responsibility for their health, producing no or few effects^60^. Educational approaches need to be appropriate not only to the individual aspects, but also to the social and interpersonal relations of the subjects.

Motivational Interviewing is a technique centered on and customized around the individual, seeking to assist him or her in the resolution of dilemmas and in the achievement of the necessary motivation for the change of health behaviors^37^. It presents a collaborative, evocative character and it respects the autonomy of the individual. When done effectively, it increases the likelihood of the individual in engaging in behavioral change. It is an alternative to traditional approach, with evidences of effectiveness for several health areas^32^ ^,^ ^43^. Regarding oral health, two systematic reviews have included a total of 18 studies investigating the effects of the Motivational Interviewing on oral health compared to the traditional educational approach (e.g., transmission of knowledge) or no approach^7^ ^,^ ^20^. The evidence is inconclusive, since the studies report controversial results, varying between positive and zero effects for the prevention of caries and periodontal disease. Despite this, the authors optimistically interpreted the potential of this approach, since there is proven effectiveness in other areas. We recommended more methodological care regarding the design, monitoring, and evaluation of these interventions in future studies in order to improve the quality of the evidence^7^ ^,^ ^20^.

The strengths of this review include the selection and evaluation of peer-reviewed articles, the inclusion of a standardized tool to evaluate the methodological quality of intervention studies, and the lack of another published review focusing on educational interventions integrated to health services, which reinforces the relevance of the results for public health. Among the limitations of this review, we can mention the non-inclusion of all existing databases and the impossibility of performing statistical synthesis using the meta-analysis given the methodological heterogeneity and the different target populations of the existing interventions.

We can conclude that the interventions performed by health professionals in the daily routine of their practice resulted in the improvement of behaviors related to oral health in most studies. All studies showed a reduction in new lesions/cases of caries, even if it was not always significant. Most studies were aimed at children; therefore, the evidence of the effectiveness of these interventions for other population groups is still unknown. In order to reinforce the evidence, future studies need to be carried out with adults and older adults, with higher methodological quality and theoretical background on the interventions, and better reporting in the publications and analysis of the results.
